# Integrative omics analysis on phytohormones involved in oil palm seed germination

**DOI:** 10.1186/s12870-019-1970-0

**Published:** 2019-08-19

**Authors:** Yong Wang, Yin Min Htwe, Jing Li, Peng Shi, Dapeng Zhang, Zhihao Zhao, Leonard Osayande Ihase

**Affiliations:** 1 Hainan Key Laboratory of Tropical Oil Crops Biology, Coconut Research Institute, Chinese Academy of Tropical Agricultural Sciences, Wenchang, 571339 People’s Republic of China; 2 Biotechnology Research Department, Ministry of Education, Kyaukse, 100301 Myanmar; 3 Nigerian Institute for Oil Palm Research (NIFOR), Benin, Nigeria

**Keywords:** Dormancy, Hormone, Inhibitor, iTRAQ, RNA-seq, Western blot

## Abstract

**Background:**

Heat treatment is widely used to break dormancy for seed germination and phytohormones could be deeply involved. However, effect of heat treatment on phytohormone related genes/proteins/metabolites and possible relationship with dormancy release remains unclear in oil palm. In this study, oil palm seeds were heat-treated at 39 °C for 60 days according to the method for commercial production. The embryos of seeds during heat treatment (0 d, 15 d, 30 d, 45 d and 60 d) and of germinated seeds (70 d and 75 d) were selected to discover the mechanisms involved in oil palm seed germination. RNA-seq and iTRAQ were applied to investigate DEGs and DEPs related to seed germination; qPCR and western blot were used as validation accordingly; endogenous phytohormones were determined by LC-MS/MS and exogenous phytohormones were also applied to validate their effects on seed germination.

**Results:**

RNA-seq results showed that plant hormone signal transduction was one of the most important pathways and eight phytohormones involved, while six of them (ABA, GA, ET, CTK, IAA and JA) were also identified by iTRAQ. Both RNA-seq and iTRAQ results showed that the expression of ABA decreased after heat treatment, which was further validated by qPCR and western blot. Furthermore, changes in endogenous phytohormones showed that ABA decreased rapidly to about 9% of the control at 30 d and then stayed at very low levels until germination; GA and CTK increased while IAA was not affected by heat treatment. Besides, exogenous ABA treatments (10, 100, 1000 mg/L) showed that the germination rate decreased to 63, 42 and 16% of the control, respectively, suggesting that ABA suppress seed germination and the inhibition effect increase with higher concentration; while the germination rates of exogenous GA and IAA treatments barely changed among different concentrations.

**Conclusions:**

Phytohormones are deeply involved in oil palm seed germination and ABA acts as an inhibitor. Heat treatment can eliminate endogenous ABA and break dormancy, while GA and CTK may also involve in dormancy release. At least 30 days of heat treatment might be necessary. This study provided informative perspectives on oil palm seed germination, which could be also applicable in other palm species.

**Electronic supplementary material:**

The online version of this article (10.1186/s12870-019-1970-0) contains supplementary material, which is available to authorized users.

## Background

Oil palm (*Elaeis guineensis* Jacq.) is one of the largest sources of vegetable oils, which contributes 33% of vegetable oil and 45% of edible oil production throughout the world [1, 2]. With the increasing population, sustainable development of this industry is necessary to meet the increasing consumption of palm oil globally [3].

Seed germination is crucial to seedling propagation and agricultural production [4–6]. Oil palm seeds are difficult to germinate because of seed dormancy and the germination is very slow under natural conditions. Heat treatment is an effective method to break dormancy for rapid germination [7–10].

Previous studies showed that the key players in the process of seed germination are plant hormones and the major phytohormones are abscisic acid (ABA) and gibberellin (GA), which have antagonistic effects on seed germination [11]. ABA is the key hormone in maintaining seed dormancy while GA is known to counteract ABA effects, thus promoting seed germination [12, 13]. ABA accumulates to initiate seed dormancy during seed maturation; ABA is down-regulated while GA is up-regulated before the onset of germination process. In addition, several other phytohormones including auxin (Aux), ethylene (ET), brassinosteroid (BR), cytokinin (CTK) and jasmonic acid (JA) are also involved in seed dormancy and germination processes [14].

Several studies have reported the effect of phytohormones on seed germination [13–17]. Furthermore, changes in hormone concentrations during dormancy release of oil palm seeds have been reported [18]. Nevertheless, previous studies on the effect of heat treatment mainly focus on changes in seed germination rate and endogenous phytohormones, related mechanism remains unclear and further systematic research through integrative omics analysis is required.

The oil palm genome (1.8 Gb) was released by the Malaysian Palm Oil Board [2] and was then widely used as the reference genome. Transcriptomic analysis has been immensely useful in discovering mRNA expression levels [19], and proteomic analysis can identify gene expression changes at protein level [20]. However, analyzing transcriptome or proteome separately may not be very informative, while integrative transcriptomic-proteomic analysis could provide complementary information [20]. For example, the integrative analysis of the transcriptome and proteome improves the understanding of fruit ripening in citrus [21], the integration of the proteome and transcriptome reveals multiple levels of gene regulation in the rice dl2 mutant [22]. Therefore, based on available oil palm genome in public database, integrative omics analysis may help to reveal more comprehensive information and related mechanism in the oil palm seed germination process. While to our knowledge, the integrative analysis of transcriptomics and proteomics in oil palm seed germination has not been reported previously.

In this study, Integrative analysis of RNA-seq and iTRAQ were applied to investigate DEGs and DEPs related to oil palm seed germination. The expression profiles of phytohormone related DEGs/DEPs at different developmental stages were validated by qPCR and western blot analysis, respectively. The changes in the endogenous phytohormones during seed germination were determined by LC-MS/MS analysis. Furthermore, exogenous phytohormones were applied to validate their effects on seed germination. As seed germination is very important for propagation, the present study may provide new perspective on oil palm seed germination, which could be useful for oil palm seedling propagation and may be also applicable in other palm species.

## Results

### Assessment digital gene expression (DGE) sequencing

Nine DGE libraries were generated from non-germinated (0 d) and germinated embryos (70 d and 75 d), with three biological replicates. Each library produced over 12 million high quality clean reads, and the percentage was above 99% among the raw reads (Additional file 1: Figure S1). Among the clean reads, the number of sequences that could be mapped to reference genome ranged from 10 to 11 million (> 85%). The percentage of unique match was > 82% and the percentage of multi-position mapped reads was < 3% (Additional file 2: Table S1).

The expression level of each gene is determined by the fragments per kilobase of transcripts per million fragments mapped (FPKM) method. The results of gene expression and related information for all samples were given in Additional file 3: Table S2. The correlation of gene expression level among biological replicates is a key criterion to test whether the experiments are reliable and whether the samples chosen are reasonable. If one sample is highly similar with another one, the correlation value between them is very close to 1. In this analysis, the correlation value between each two samples was calculated based on FPKM result. All the correlation values are ≥0.99, indicating a good correlation between replicates (Additional file 4: Table S3).

### Differentially expressed genes (DEGs) and proteins (DEPs) analysis

**Fig. 1:**
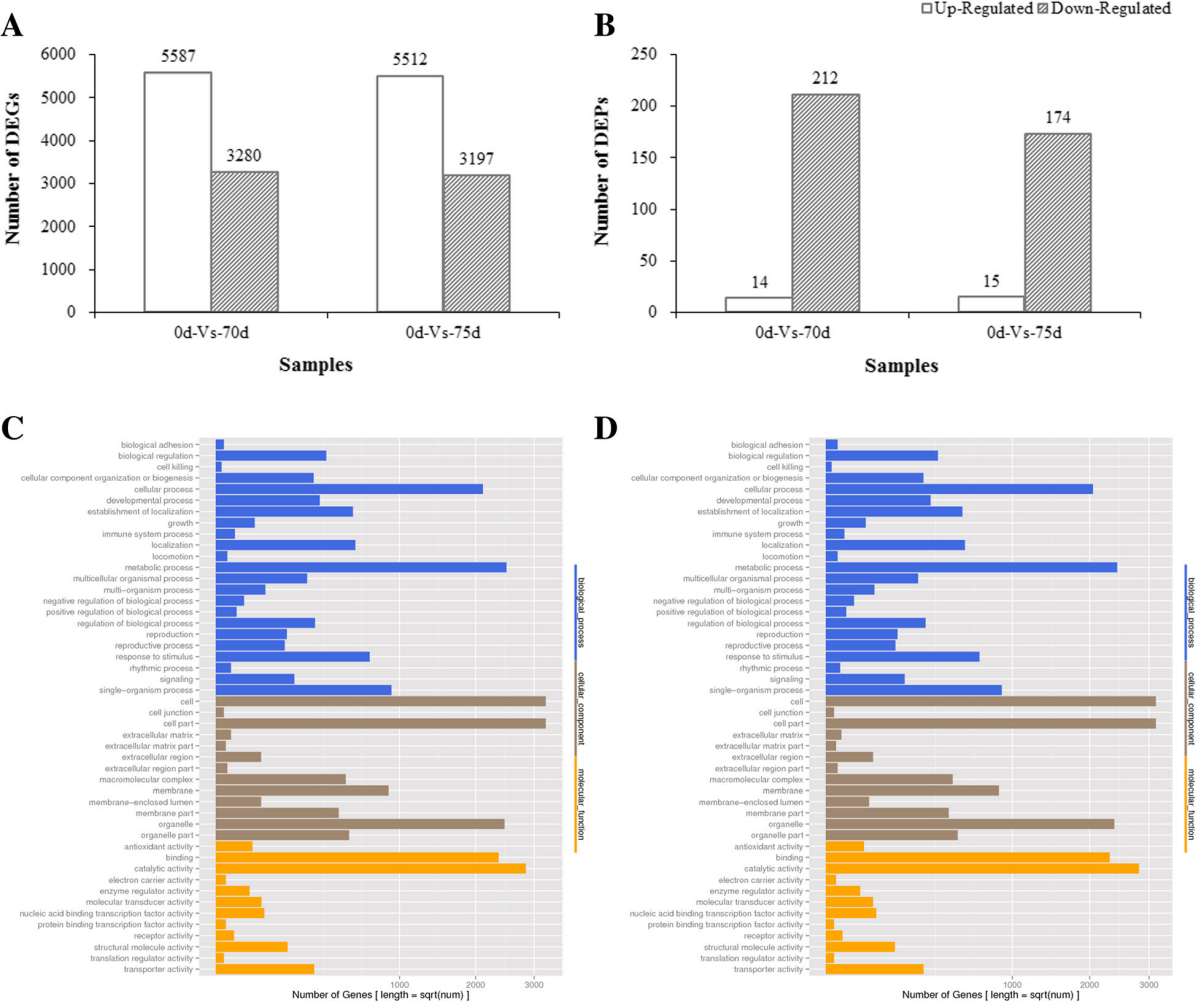
**a**-**b** Statistics of DEGs and DEPs. **c** GO classification of DEGs in 0d-Vs-70d and (**d**) GO classification of DEGs in 0d-Vs-75d

The differentially expressed genes (DEGs) and differentially expressed proteins (DEPs) were identified in (a) between 0d and 70d; (b) between 0d and 75d. Totally 8867 DEGs were identified in 0d-Vs-70d, in which 5587 and 3280 genes were up-regulated and down-regulated, respectively. Totally 8709 genes were identified as DEGs in 0d-Vs-75d, in which 5512 genes were up-regulated and 3197 genes were down-regulated. The number of DEGs identified in 0d-Vs-70d was a little bit higher than 0d-Vs-75d and more DEGs were up-regulated (~ 63%) than down-regulated in both comparisons (Fig. 1a, Additional file 5: Table S4). These genes were associated with several gene families such as plant hormone related genes (abscisic acid receptor PYR/PYL family, gibberellin receptor, AUX1 LAX family, ethylene response factor, cytokinin receptor and others), transcription factors, cytochrome P450, heat shock protein and so on (Additional file 5: Table S4). The expression of some genes was highly significant with log2Ratio ≥ 10. A list of some DEGs with log2Ratio ≥ 10 was shown in Additional file 6: Table S5.

A total of 4592, 3371 and 3447 proteins were identified in three biological replicates, respectively (Additional file 7: Table S6). As shown in Additional file 8: Figure S2, a total of 7413 proteins were identified by merging the data obtained from three biological replicates. Totally 2178 and 2182 proteins were detected in 0d-Vs-70d and 0d-Vs-75d, respectively. In 0d-Vs-70d, 226 proteins were identified as DEPs, in which 14 DEPs were up-regulated and 212 DEPs were down-regulated. As for 0d-Vs-75d, 189 proteins were identified as DEPs, in which 15 DEPs were up-regulated and 174 DEPs were down-regulated. However, unlike DEGs, the majority of DEPs were down-regulated in both 0d-Vs-70d (94%) and 0d-Vs-75d (92%) (Fig. 1b, Additional file 9: Table S7).

### GO classification and KEGG pathway enrichment of DEGs

DEGs were categorized into biological process, cellular component and molecular function using BLAST2GO program. According to GO classification, the main groups of each category in 0d-Vs-70d and 0d-Vs-75d were very similar as follows: main biological processes were metabolic and cellular; major cellular components were cell, cell part and organelle; major molecular function were catalytic activity and binding (Fig. 1c and d). In addition, DEGs were also involved in hormone stimulus, hormone mediated signaling pathway, hormone metabolic process, abscisic acid stimulus, gibberellins metabolic process and other plant hormone related processes (Additional file 5: Table S4), which revealed that plant hormone play crucial role in response to heat stress and break dormancy.

According to KEGG pathway enrichment, 5242 DEGs in 0d-Vs-70d and 5130 DEGs in 0d-Vs-75d were enriched into 126 pathways, respectively. Top 3 pathways were same (Additional file 10: Figure S3) as follows: Metabolic pathways (1267 and 1251 DEGs, respectively), Biosynthesis of secondary metabolites (726 and 725 DEGs, respectively), Plant hormone signal transduction (268 and 266 DEGs, respectively). As for plant hormone signal transduction pathway (Table 1), 268 DEGs in 0d-Vs-70d and 266 DEGs in 0d-Vs-75d, respectively, were involved in 8 plant hormones (Aux, CTK, GA, ABA, ET, BR, JA and SA). The unigenes involved in plant hormone signal transduction pathway and their expression levels were given in Additional file 11: Table S8.

**Table 1:** DEGs involved in plant hormone signal transduction pathway

Components	0d-Vs-70d			0d-Vs-75d		
	DEGs	↑regulated	↓regulated	DEGs	↑regulated	↓regulated
Auxin	45	36	9	41	32	9
AUX1	5	5	0	4	4	0
TIR1	5	3	2	3	2	1
AUX/IAA	14	12	2	14	11	3
ARF	9	6	3	9	6	3
GH3	3	3	0	3	3	0
SAUR	9	7	2	8	6	2
Cytokinin	40	30	10	41	30	11
CRE1	19	12	7	18	11	7
B-ARR	18	15	3	20	17	3
A-ARR	3	3	0	3	2	1
Gibberellin	27	23	4	27	23	4
GID1	11	10	1	12	11	1
GID2	2	2	0	2	2	0
DELLA	14	11	3	13	10	3
Abscisic acid	43	31	12	45	33	12
PYR/PYL	8	8	0	8	8	0
PP2C	30	21	9	31	22	9
SnRK2	1	0	1	2	1	1
ABF	4	2	2	4	2	2
Ethylene	28	15	13	30	15	15
ETR	2	0	2	2	0	2
CTR1	13	7	6	12	5	7
MPK6	3	3	0	3	3	0
EIN2	4	0	4	4	0	4
EBF 1/2	0	0	0	1	0	1
ERF 1/2	6	5	1	8	7	1
Brassinosteroid	42	33	9	37	30	7
BAK1	13	10	3	10	7	3
BRI1	9	7	2	8	7	1
BSK	1	1	0	1	1	0
BSU1	1	0	1	0	0	0
BZR1/2	6	4	2	6	4	2
TCH4	7	6	1	8	7	1
CYCD3	5	5	0	4	4	0
Jasmonic acid	30	26	4	32	29	3
JAR1	2	2	0	2	2	0
COI1	1	0	1	1	0	1
JAZ	11	11	0	11	11	0
MYC2	16	13	3	18	16	2
Salicylic acid	13	9	4	13	10	3
NPR1	2	2	0	1	1	0
TGA	10	6	4	11	8	3
PR1	1	1	0	1	1	0
Total	268	203	65	266	202	64

### GO classification and KEGG pathway enrichment of DEPs

**Fig. 2:**
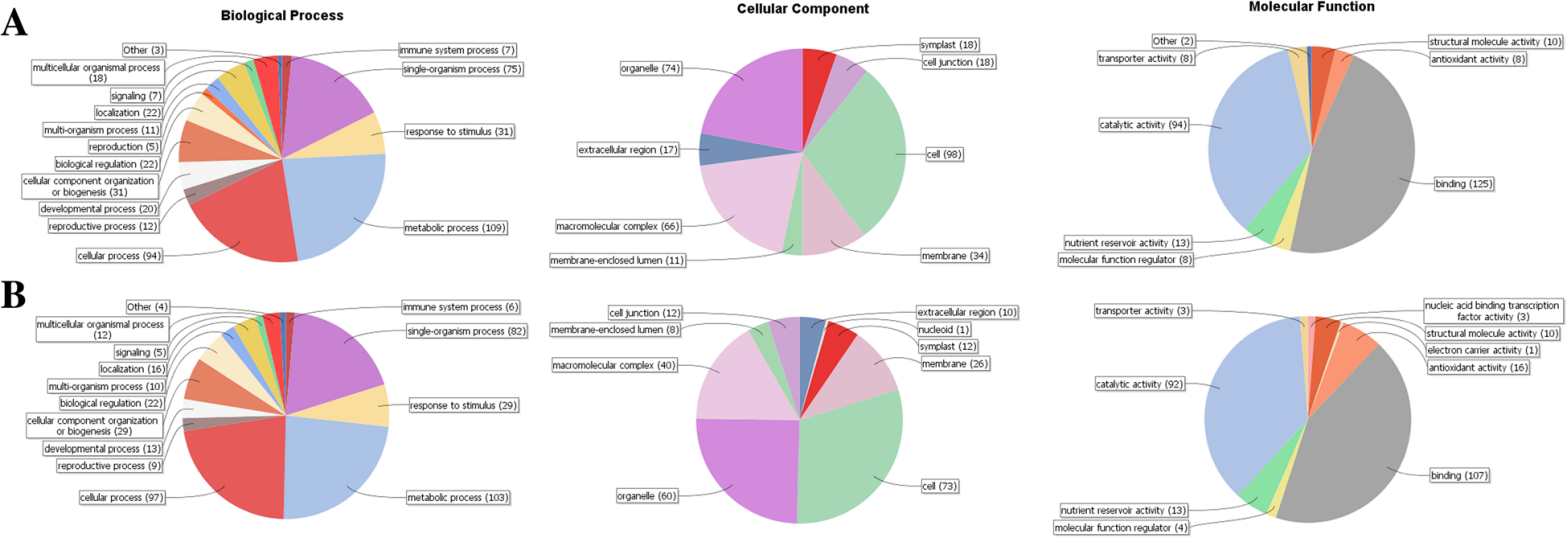
GO classification of DEPs. a 0d-Vs-70d. b 0d-Vs-75d

DEPs were subjected to GO classification and were categorized into biological process, cellular component and molecular function. Quite similar to DEGs, the main biological processes represented in 0d-Vs-70d were metabolic process (109) and cellular process (94), major cellular components were cell (98) and organelle (74), main molecular functions were binding (125) and catalytic activity (94) (Fig. 2a). Very similar results were also showed in 0d-Vs-75d (Fig. 2b).

As compared with all identified proteins, percentage of differential proteins in 0d-Vs-70d showed significant change in 368 GO categories while that in 0d-Vs-75d showed significant change in 485 GO categories. GO term with FDR ≤ 5% was considered to be significantly enriched (Additional file 12: Figure S4 and Additional file 13: Table S9).

**Fig. 3:**
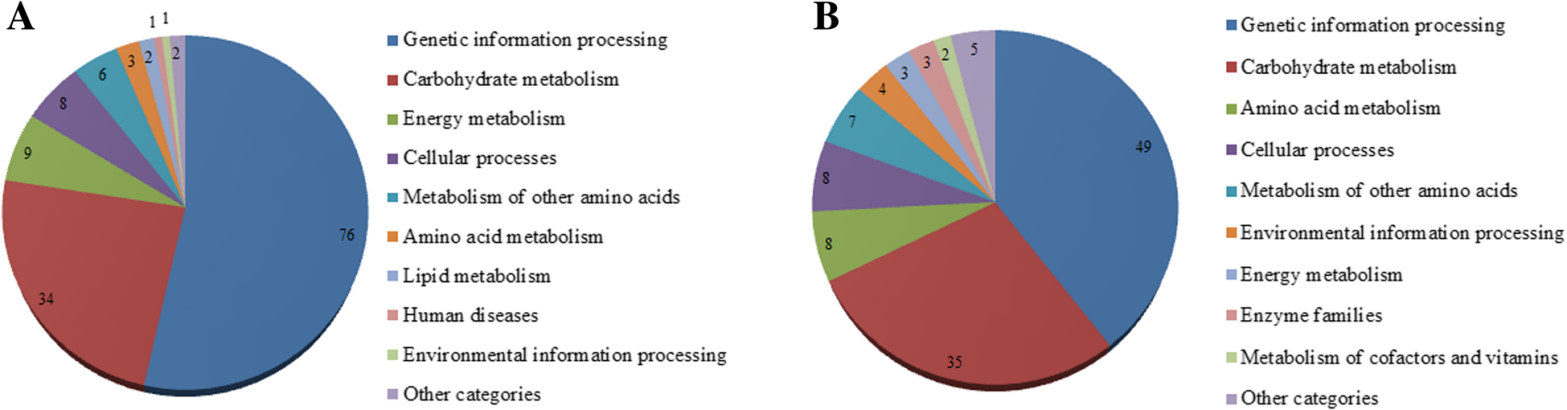
KEGG pathway enrichment of DEPs. a 0d-Vs-70d. b 0d-Vs-75d

According to KEGG pathway enrichment, 142 DEPs were enriched into 10 pathways in 0d-Vs-70d, the major pathways were genetic information processing (76) and carbohydrate metabolism (34) (Fig. 3a). Similarly, 124 DEPs were enriched into 10 pathways in 0d-Vs-75d, the major pathways were also genetic information processing (49) and carbohydrate metabolism (35) (Fig. 3b).

### Expression profiles of phytohormone related DEGs/DEPs

**Fig. 4:**
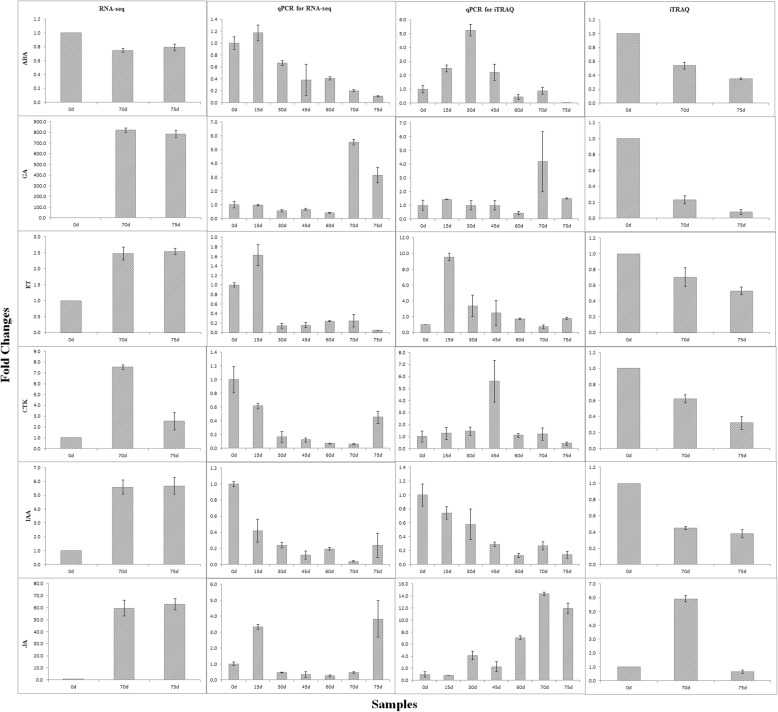
Expression profiles of phytohormone related DEGs and DEPs

To investigate these identified phytohormones, their expression profiles in RNA-seq and iTRAQ analysis, as well as that at mRNA levels by qPCR, were put together in Fig. 4. According to RNA-seq analysis, ABA was down-regulated while other phytohormones (GA, ET, CTK, IAA and JA) were up-regulated at germinated stages (70 d and 75 d), which were also supported by qPCR results except for that of ET, CTK and IAA. Meanwhile, according to iTRAQ analysis, all of these phytohormones were down-regulated at germinated stages except that JA was up-regulated at 70 d and then down-regulated again at 75 d. While these results were only supported by corresponding qPCR results of ABA and IAA, in which ABA was up-regulated first before 30 d might result from the heat shock response.

In summary, comprehensive results of RNA-seq, iTRAQ and qPCR analysis indicated that ABA might play a key role during oil palm seed germination.

### Expression profiles of target proteins by western blot

**Fig. 5:**
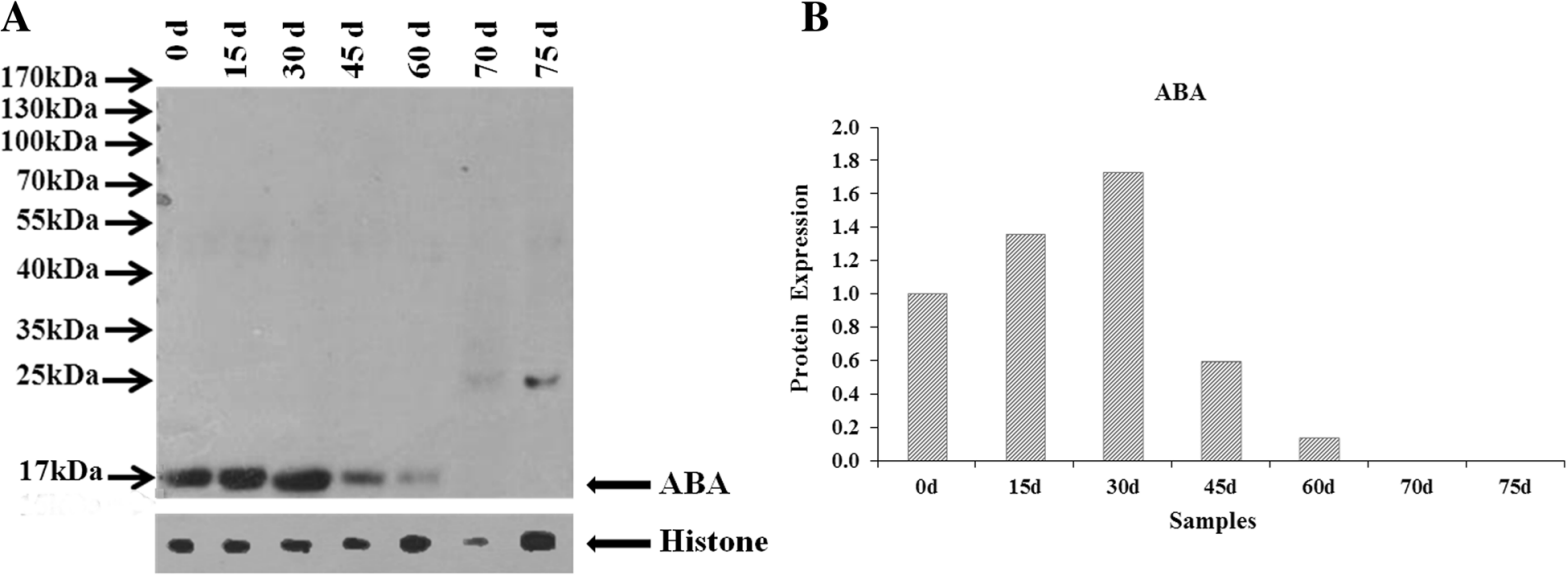
Western blot validation. a Western blot for ABA antibody. b Expression profiles of ABA related protein

Antibodies for six phytohormone related proteins were synthesized and prepared, but only one antibody (28786-1hz-2/C3) for ABA (ACF06553.1) was successful for validation. The other antibodies for GA (28788-1hz-10/C13), ET (28789-1hz-2/C5), CTK (28790-1hz-12/C16), IAA (28791-1hz-9/C19), and JA (28785-1hz-10/C14) were failed to synthesize. The western blot results showed that the antibody for ABA has a specific band at about 17 kDa in samples 0 d, 15 d, 30 d, 45 d and 60 d (Fig. 5a). Normalization results (Table 2) showed that ABA highly expressed in early stages until 30 d and then decreased dramatically to 14% of the control at the end of heat treatment (60 d), further decreased to 1% of the control at the beginning of seed germination (70 d) and completely disappeared at 75 d (Fig. 5b).

**Table 2:** Western blot analysis

Samples	0 d	15 d	30 d	45 d	60 d	70 d	75 d
ABA	17.79	25.28	31.15	10.62	3.89	0.06	0.07
Histone	9.06	9.49	9.22	9.14	14.39	4.51	25.18
ABA/Histone	1.96	2.66	3.38	1.16	0.27	0.01	0.00
Normalization	1.00	1.36	1.72	0.59	0.14	0.01	0.00

Note: Values represent protein expression levels. Expression of each sample was divided by ABA/Histone (1.96) for normalization

### Changes in endogenous phytohormones during seed germination

**Fig. 6:**
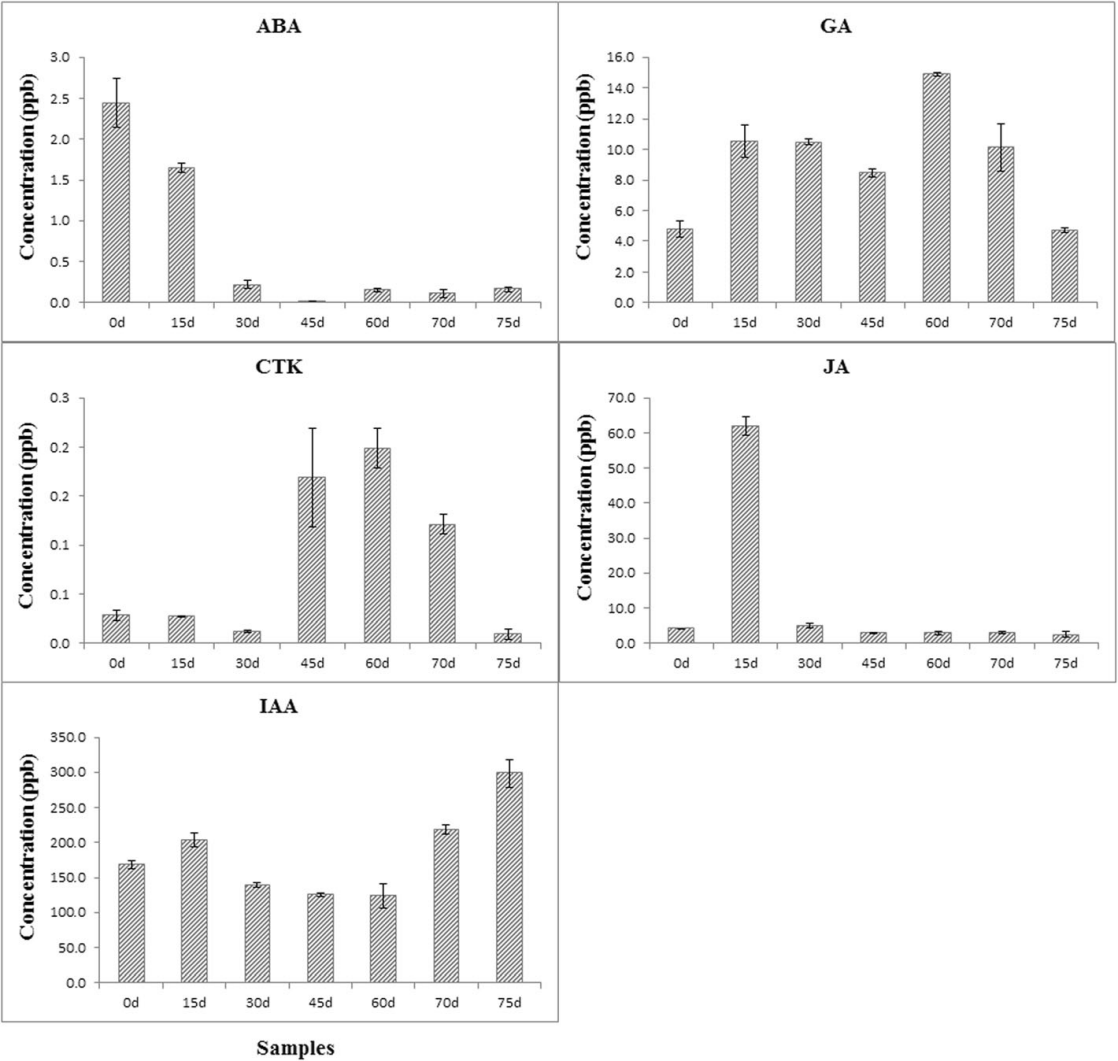
Changes in endogenous phytohormones during seed germination

The concentration of endogenous phytohormones (ABA, GA, ET, CTK, IAA, and JA) in the embryos during seed germination was determined using LC-MS/MS system. Results showed that endogenous ABA decreased rapidly to about 9% of the control at 30 d and then stayed at very low levels until germination. GA responded rapidly since the very beginning of heat treatment, it increased till the end of heat treatment (60 d) and then decreased to approximately that of the control level at 75 d. While CTK hardly changed at the beginning of heat treatment, it increased dramatically at 45 d and 60 d, and then similar decreases to GA were also observed since germination (70 d), suggesting that endogenous GA and CTK experienced similar changes as a result of heat treatment. While JA was not evident throughout the process except for a sudden rise at 15 d. As for IAA, it barely changed during heat treatment and then increased rapidly along with germination since 70 d (Fig. 6), indicating that the heat treatment has no effect on endogenous IAA. These results suggested that heat treatment can eliminate endogenous ABA and oil palm seeds are able to germinate only after endogenous ABA decreased to a very low level by heat treatment. At least 30 days of heat treatment is necessary to break dormancy.

### Effect of exogenous phytohormones on seed germination

**Fig. 7:**
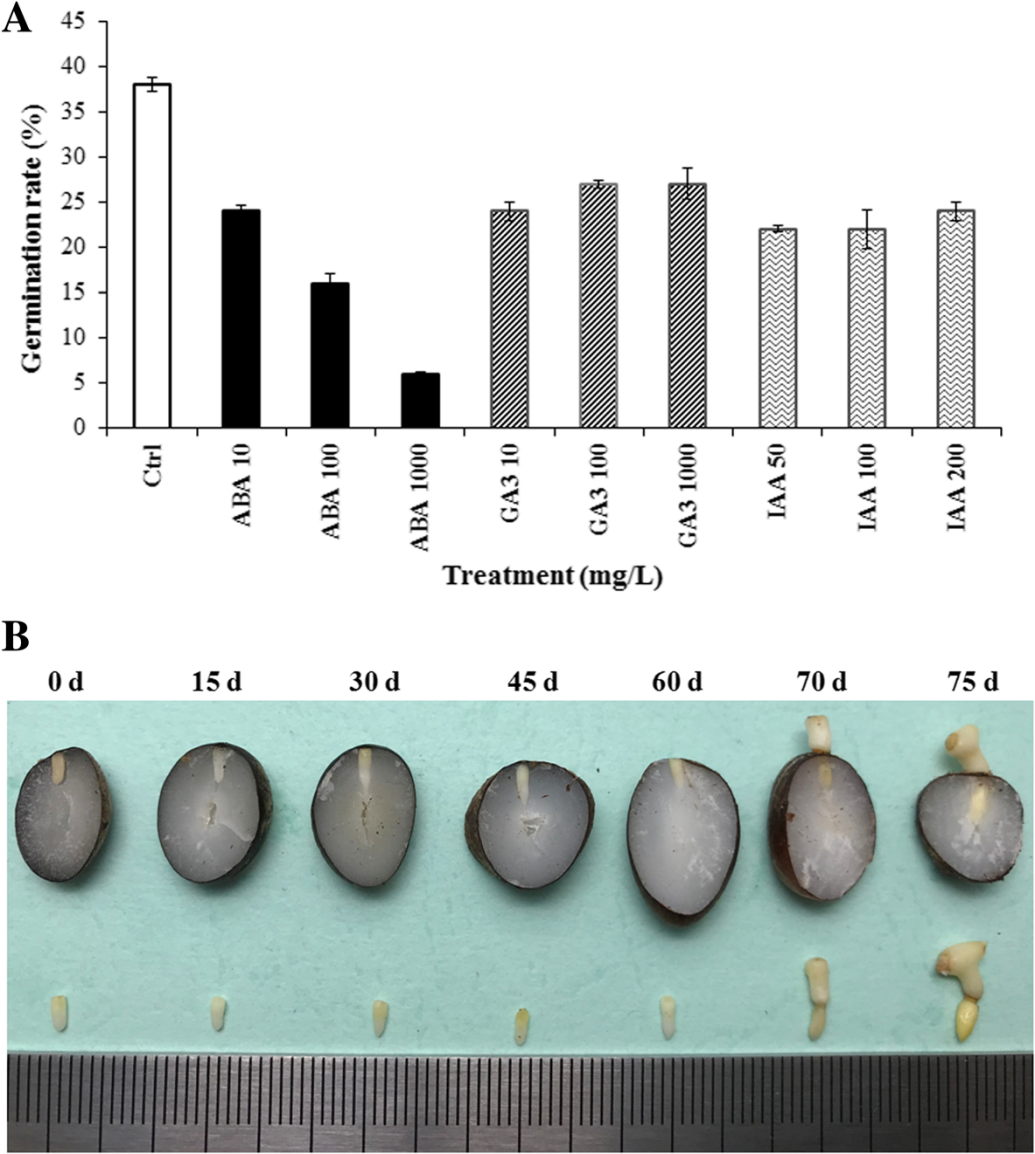
**a** Effect of exogenous phytohormones on seed germination. The treatment with distilled water was taken as control (Ctrl) and 100 oil palm seeds were used in each treatment. **b** Germination of oil palm seeds

Seed germination results showed that germination rates of all exogenous treatments were lower than the control (38%). Remarkably, with the increasing concentration of ABA treatments, the germination rate decreased rapidly from 24% (10 mg/L) to 16% (100 mg/L) and 6% (1000 mg/L), which was only 63, 42 and 16% of the control, respectively, indicating that exogenous ABA could inhibit seed germination again even after endogenous ABA already decreased to a very low level by heat treatment. However, germination rates of exogenous GA and IAA treatments barely changed among different concentrations (Fig. 7a).

## Discussion

Oil palm seeds are difficult to germinate under natural conditions. Heat treatment is an effective method to break dormancy for rapid germination [10] and phytohormones are also known as one of the key players that control seed dormancy and germination [11, 23]. However, related mechanism stays unclear in oil palm.

In this study, RNA-seq and iTRAQ analysis were applied to investigate DEGs and DEPs related to seed germination. RNA-seq analysis identified 8867 and 8709 DEGs in 0d-Vs-70d and 0d-Vs-75d, while iTRAQ analysis identified 226 and 189 DEPs in 0d-Vs-70d and 0d-Vs-75d, respectively. More DEGs were up-regulated (~ 63%) in RNA-seq while the majority of DEPs were down-regulated (~ 93%) in iTRAQ. In general, the major categories resulted from GO classification of both DEGs and DEPs were very similar as follows: metabolic and cellular in biological processes, cell and organelle in cellular components, catalytic activity and binding in molecular function. However, the expression profiles of some identified phytohormones at mRNA levels were not completely in agreement with that at protein levels (Fig. 4). This may be due to post-transcriptional and post-translational regulating mechanisms [21, 22, 24].

According to RNA-seq analysis, over 260 DEGs related to 8 phytohormones were involved in plant hormone signal transduction pathway (ko04075), and 6 of them (ABA, GA, ET, CTK, IAA and JA) were also identified by iTRAQ, indicating that phytohormones are deeply involved in oil palm seed germination process.

Endogenous ABA is the key factor result in oil palm seed dormancy. Both RNA-seq and iTRAQ results showed that ABA decreased after heat treatment, and corresponding validations by qPCR and western blot also revealed the decrease of endogenous ABA. Although ABA increased first at early stages during heat treatment in the present study, it might result from the heat shock response, which could be always related to rapid increase of endogenous ABA in crop plants under abiotic stress [25, 26]. Besides, the most direct evidence resulted from the determination of endogenous phytohormones, in which ABA decreased rapidly to only about 9% of the control at 30 d and remained at very low levels thereafter till germination. These results suggested that heat treatment could eliminate endogenous ABA to break seed dormancy and at least 30 days of heat treatment might be necessary, which was in agreement with previous results in oil palm [18]. Studies in other crops also gave similar results. ABA could positively regulate seed dormancy while GA could break seed dormancy and induce germination [27, 28]; ABA accumulated during seed maturation to initiate dormancy, however, it was down-regulated while GA was up-regulated before the onset of germination process [14]. Furthermore, the seed germination after heat treatment was highly suppressed again by exogenous ABA treatments at the imbibition stage (Fig. 7a), which confirmed the crucial role of ABA. The oil palm seeds could be able to germinate only after endogenous ABA was almost completely eliminated by heat treatment.

Endogenous GA and CTK may antagonize ABA suppression to seed germination. ABA decreased rapidly since the very beginning of heat treatment and almost disappeared since 30 d; while on the contrary, both GA and CTK increased by heat treatment, suggesting that endogenous GA and CTK may antagonize ABA to break seed dormancy. Previous study also showed that cytokinin antagonizes ABA suppression to seed germination of Arabidopsis by down-regulating ABI5 expression [29]. However, Arabidopsis mutants without CTK receptors had more rapid seed germination [30], indicating that CTK may inhibit germination and further detailed investigations are still needed. On the other hand, endogenous GA and CTK decreased since germination (70 d), suggesting that they might be further involved in embryo development after successful germination. As for JA, it was not evident throughout the germination except for a sudden rise at 15 d, suggesting that JA may be not important in oil palm seed germination process. In summary, endogenous ABA is responsible for seed dormancy, while GA and CTK may be related to dormancy release.

Recent study [31] revealed that ABA levels in the rice seeds stressed at 35 °C decreased with increasing time of heat stress, while GA3 levels increased as compared with non-stressed seeds. Their results are in agreement with our results, suggesting that hormonal response to heat stress observed in oil palm is similar with rice.

Endogenous IAA in oil palm seeds was not obviously affected by heat treatment. Although IAA increased rapidly along with germination since 70 d (Fig. 6), it might result from the vigorous growth of germinated embryos, because Auxin involved in almost all aspects of plant development including shoot and root developments [14]. However, exogenous IAA could inhibit seed germination. Previous study showed that exogenous IAA could delay seed germination as the complement of ABA in preharvest sprouting of wheat [32]. Similarly, earlier studies in *Arabidopsis* reported that exogenous auxin could enhance the inhibition on seed germination by ABA, but cannot inhibit seed germination in the absence of ABA [5, 33, 34]. Therefore, endogenous IAA in oil palm seeds might not be affected by heat treatment while exogenous IAA could inhibit seed germination.

## Conclusions

In conclusion, integrative omics analysis using RNA-seq and iTRAQ along with validations by qPCR, western blot, endogenous quantification of phytohormones and exogenous phytohormones treatments, revealed that phytohormones are deeply involved in oil palm seed germination and ABA act as an inhibitor, similarly as described in most plant species. Heat treatment improves oil palm seed germination by eliminating endogenous ABA and breaking dormancy, while GA and CTK may also involve in dormancy release. At least 30 days of heat treatment is necessary to break dormancy. These results could be useful for oil palm seedling propagation and may be also applicable in other palm species.

## Methods

### Embryo preparation

Oil palm seeds were collected from the oil palm germplasm nursery in Coconut Research Institute, Chinese Academy of Tropical Agricultural Sciences, Wenchang, China. Dry heat method was used for seed germination according to Corley [8] as follows: The seeds were dried naturally under the shade to reach the moisture content of 18% and kept in tightly sealed polythene bags, and then were placed in the germinator at 39 °C to break dormancy (heat treatment). During heat treatment, seeds were collected at 15 d, 30 d, 45 d, and 60 d, respectively. Thereafter, the seeds were soaked in distilled water for about 2 days to reach the moisture content of 22% and then placed at 27 °C for germination. The seeds were then sectioned and embryos were collected from both non-germinated (15 d, 30 d, 45 d, 60 d) and germinated seeds (70 d, 75 d). Embryos of untreated seeds (0 d) were collected before treatment as the control and kept in freezer at − 80 °C. About 2 g of embryos from each sample (0 d, 15 d, 30 d, 45 d, 60 d, 70 d and 75 d) were collected (Fig. 7b) and frozen in liquid nitrogen immediately. Thereafter, they were kept in freezer at − 80 °C for the following analyses.

For RNA-seq and iTRAQ analysis, embryos of control (0 d) and germinated (70 d and 75 d) were selected, while the rest embryos (15 d, 30 d, 45 d, 60 d) without obvious morphological changes during the heat treatment process were not used. The experiment included three biological replicates for each stage.

### RNA extraction and RNA-seq analysis

Total RNA of each sample was extracted using Trizol Reagent (invitrogen, USA) according to the manufacturer’s instruction and treated with DNaseI (Takara, Japan) to degrade any possible DNA contamination. Concentration and purity of each RNA sample was determined using ND-2000 spectrophotometer (NanoDrop, USA), and RNA integrity was examined by 1.5% agarose gel electrophoresis. Identical RNAs of each sample were used for RNA-seq analysis and the following qPCR validation.

Total RNA of embryos (0 d, 70 d and 75 d) were submitted to BGI (Shenzhen, China) for RNA-seq analysis. The library was constructed and then sequenced using Illumina Hiseq 2000 by BGI (Shenzhen, China). After sequencing, raw reads containing low quality reads, reads with adapters, and reads with unknown bases more than 10% were filtered before data analysis. Clean Reads were used for downstream bioinformatics analysis. The clean reads were mapped to reference genome using BWA [35]. Gene expression level was quantified using RNA-seq by expectation maximization (RSEM) [36]. The gene expression levels were calculated by fragments per kilobase of transcripts per million fragments mapped (FPKM). Differentially expressed genes between two groups were detected with Noiseq method [37]. In this analysis, differentially expressed genes were screened with the foldchange ≥2 and probability ≥0.8. Then, Gene Ontology (GO) classification and Kyoto Encyclopedia of Genes and Genomes (KGGG) pathway enrichment of differentially expressed genes (DEGs) were analyzed to annotate the DEGs.

### Protein extraction and iTRAQ analysis

Proteins were extracted with NitroExtra™ (Cat. PEX-001–250ML, N-Cell Technology, China). Sample debris were removed by high centrifugation at 100,000×g, using SW-28 rotor for 2 h at 10 °C. After centrifugation, proteins were precipitated with 1:3 (Sample to Acetone) cold acetone at − 20 °C overnight. Precipitated proteins were washed twice with cold acetone and finally re-suspended in 8 M urea after protein precipitate had been air dried.

Proteins of embryos (0 d, 70 d and 75 d) were submitted to N-Cell Technology (Shenzhen, China) for iTRAQ analysis. Proteins were alkylated and then determined by DC Assay Kit (Cat. 500–0111, BioRad). Trypsin digestion was performed in 100 mM TEAB (pH 8, T7408, Sigma) and the trypsin digest was incubated at 37 °C for 18 h. Digested proteins were desalted by C18 column and dried in spin vacuum. Then 100 μg of desalted peptides from each sample was chemically labeled with iTRAQ 8-plex reagent (Cat. 4,466,096) in 100 mM TEAB according to manufacturer’s protocol. After desalting, differentially labeled peptide samples were re-suspended in SCX for peptide fractionation. Totally 53 of 1 mL fractions were collected and further combined into 10 fractions based on the number of proteins identified in each 5 min fraction. Each fraction was desalted with ZipTip (Cat. ZTC18S960, Millipore) and dried in spin vacuum for LC-MS/MS analysis by AB SCIEX 6600 (USA).

Raw data files were transformed into Mascot Generic Format (MGF) and mzXML format using OpenMS. The MGF files were searched against the NCBI and common MS contaminant database using Mascot (Matrix Science) software. The tolerance for MS1 and MS2 error was 20 ppm and 50 mmu respectively. Caramidomethylation (+ 57 Da) was added as fixed modification while iTRAQ 8-Plex (K/N) and Oxidation (M) were added as variable modifications. A maximum of 2 trypsin miss cleavages were allowed. The instrument type was chosen as ESI-QTOF. The mass input was assumed to be monoisotopic mass. Decoy database was used for the estimation of false discovery rate (FDR). The protein ratios of *P* < 0.05 with 5% FDR correction were filtered as differential proteins for following analyses.

### qPCR validation

Complementary DNA (cDNA) from total RNA was prepared using RevertAid™ first strand cDNA synthesis kit (Fermentas, Lithuania) according to the manufacturer’s protocol. Phytohormone related DEGs/DEPs were selected from RNA-seq/iTRAQ for expression validation after protein sequences were translated to nucleotide sequences using NCBI. Primers (Tables 3 and 4) were designed with Primer-BLAST and synthesized by BGI (Shenzhen, China). PCR optimization was performed in PCR Thermal Cycler Dice™ TP600 (TaKaRa, Japan).

**Table 3:** Target genes from RNA-seq and corresponding primers for qPCR

Name	Gene ID	Primer sequences (5′-3′)	Amplicon length (bp)
ABA	XM_010922729.2	F- TGGTGGAATCGTACGTGGTC R- CACTTGACGATTGTGTCCGC	82
GA	XM_010921546.2	F- AGCCAACCCACTAGACCAAA R- ACAGCCTCCTTCTCCAAGTCT	198
ET	JN203269.1	F- TTGACTCAGGCACAACTTGC R- GCCAGCTACGATTAGTTTCCCT	118
CTK	NM_001156290.2	F- GACGTGCCACTTCACAATGG R- CCCTCACCACCCAAAGGAAT	86
IAA	KC146057.1	F- AGGGGCTACGTGCTAGAGAA R- TTTTAATGGTGGCGCGTGTG	186
JA	NM_001055402.1	F- ATAGGGCAGTCGGCCAATAC R- CGTGGGGGCTGCTTTGC	103

**Table 4:** Target proteins from iTRAQ and corresponding primers for qPCR

Name	Protein ID	Primer sequences (5′-3′)	Amplicon length (bp)
ABA	XP_010918764.1	F- GTTTGGAGGAAGCCGATCCA R- TGAACACCAGGTGCAACAGA	127
GA	XP_010904836.1	F- CTCCTTTCGGCGAGATTGGT R- CTCTATCTCAAGCCGGGCAC	71
ET	XP_010915532.1	F- TCCCCCATCCAAAATCCAAACA R- ATTTGTACTTCGTGCCCCCTT	113
CTK	XP_010906323.1	F- TCGGCAAAAGATGCTTGGGA R- CCAGACCCTTCTTACGCTACA	79
IAA	XP_010917603.1	F- AGGAGAATGGAAGGTGGAGGA R- CAAGCCTTAGCTCGGTTGCT	70
JA	XP_010908911.1	F- AACAGGGCCTCACTCTGGTA R- GAACCGGGCTAATGATGCCT	193

The qPCR was performed on ABI-7900HT (Applied Biosystems, USA) using PowerUp SYBR Green Master Mix (Applied Biosystems, USA) under the following conditions: 95 °C for 10 min, followed by 40 cycles at 95 °C for 15 s and 60 °C for 1 min in 384 well optical plates. Each reaction mixture was 10 μL containing 1 μL of 50 ng/μL cDNA, 5 μL of 2X PowerUp SYBR Green Master Mix, 0.5 μL of each 10 μM forward and reverse primers. The raw data were analyzed using ABI-7900HT software and housekeeping gene Actin was used as an endogenous control for normalization of gene expression. Comparative Ct method was used for qPCR normalization and each sample was detected in four replicates.

### Antibody preparation and western blot validation

According to protein sequence of each phytohormone related DEP (ABA, GA, ET, CTK, IAA and JA), ten peptides were designed and synthesized, respectively, and then were injected to rabbits for antibody preparation by Abmart Biomedicine (Shanghai, China). Only successful antibodies were used for western blot validation.

SDS-PAGE and immunoblotting were performed according to standard procedures. Cell membranes were incubated with antisera that were used against one of the following individual proteins: C-24 sterol reductase (ERG4), HMG-CoA synthase (ERG13), C-4 sterol methyl oxidase (ERG25), and Actin (each 1:1000 dilution). ERG4 and ERG25 were customized from Abmart Biomedicine (Shanghai, China). ERG13 antibody was purchased from LifeSpan Biosciences (Seattle, USA) and Actin antibody was purchased from Agrisera (Vännäs, Sweden). The secondary antibody was goat anti rabbit IgG (1:10,000) conjugated with alkaline phosphatase. The bands were visualized by a premixed BCIP/NBT substrate solution (Sigma-Aldrich, USA). Histone antibody was used as control.

### Determination of endogenous phytohormones

Endogenous phytohormones were analyzed using the XEVO TQ-S system (WATERS, USA) with a chromatographic column (ACQUITY UPLC BEH C18). The fine powder (0.2 g) of each sample was soaked in 1.5 mL of extraction buffer (Methanol: Ultrapure water: Methanolic acid = 15: 4: 1). After vortexing at 60 HZ for 1 min, samples were centrifuged at 10000 rpm for 15 min at 4 °C. Then, 1 mL of supernatant was collected and filtered through a 0.22 μm filter membrane. Finally, each sample was transferred into a 2 mL LC/MS glass vial for LC-MS/MS analysis. The mobile phases and gradient were as follows: mobile phase A, Methanol; mobile phase B, Ultrapure water. The flow rate was set to 0.4 mL/min. The gradient program was as follows: 25% A and 75% B for 0 min; 25% A and 75% B for 0.5 min; 2% A and 98% B for 0.51 min; 2% A and 98% B for 1 min; 100% A for 1.5 min; 100% A for 2 min; 25 and 75% B for 3 min; 25% A and 75% B for 4 min. Multiple reaction monitoring (MRM) detection method was used for the quantification of all analytes. The mass spectrum parameters are as follows: capillary voltage, 2.0 kV; capillary temperature, 380 °C; sheath gas flow rate, 60 L/N; Aux gas flow rate, 600 L/N; MSMS collision gas, argon; collision gas flow rate, 0.18 mL/min. Each sample was extracted three times. The chemical standards of ABA (CAS 21293298), GA (CAS 77065), CTK (CAS 525791), IAA (CAS 87514) and JA (CAS 39924522) were purchased from Sigma-Aldrich (USA) and used to create calibration curves.

### Validation with exogenous phytohormones

Three phytohormones (ABA, GA, IAA) identified from this work were applied individually to validate the effect of exogenous phytohormones on seed germination. The same dry heat method was used for seed germination according to Corley [8]. After heat treatment for 60 days to break dormancy, seeds were soaked in distilled water for 2 days along with phytohormone treatments as follows: ABA (10 mg/L, 100 mg/L and 1000 mg/L), GA3 (10 mg/L, 100 mg/L and 1000 mg/L) and IAA (50 mg/L, 100 mg/L and 200 mg/L). The treatment with distilled water was taken as control (Ctrl) and 100 oil palm seeds were used in each treatment. Germination rates were recorded up to 20 days after phytohormone treatment until no more germinated seeds. The seed germination rate of each treatment was calculated using the following formula:

Germination rate (%) = (number of germinated seeds/number of treated seeds) × 100.

### Data analysis

The software SAS statistical program version 9.4 was used to analyze the data. Data in the figures were expressed as the means of all replicates ± standard deviations. The top 10 KEGG pathways and the number of DEGs enriched in each pathway were presented by Circos software [38].

## Additional files

Additional file 1: **Figure S1.** Quality assessment of reads. (DOCX 498 kb)

Additional file 2: **Table S1.** Summary of reads mapped to reference genome. (XLS 25 kb)

Additional file 3: **Table S2.** The quantification of gene expression. (XLSX 4617 kb)

Additional file 4: **Table S3.** Correlation analysis between replicates. (XLSX 11 kb)

Additional file 5: **Table S4.** Analysis of differentially expressed genes (DEGs). (XLSX 2492 kb)

Additional file 6: **Table S5.** A list of some differentially expressed genes (DEGs) with log2Ratio ≥ 10. (XLSX 45 kb)

Additional file 7: **Table S6.** Total identified proteins of three biological replicates. (XLS 12639 kb)

Additional file 8: **Figure S2.** Venn diagram of identified proteins in three biological replicates. (DOCX 181 kb)

Additional file 9: **Table S7.** Analysis of differentially expressed proteins (DEPs). (XLS 376 kb)

Additional file 10: **Figure S3.** Top 10 KEGG pathway enrichment of DEGs. Red ribbons indicate the links between 0d-Vs-70d and top 10 pathways while orange ribbons indicate the link between 0d-Vs-75d and top 10 pathways. The number of DEGs in each pathway was represented by the thickness of the ribbons. (DOCX 429 kb)

Additional file 11: **Table S8.** Differentially expressed genes (DEGs) in Plant hormone signal transduction pathway. (XLSX 21 kb)

Additional file 12: **Figure S4** Top 10 enriched GO categories of differential proteins in (A) 0d-Vs-70d (B) 0d-Vs-75d as compared with all identified proteins. (DOCX 102 kb)

Additional file 13: **Table S9.** GO classification analysis of DEPs as compared with all identified proteins. (XLSX 103 kb)

## Data Availability

RNA-seq data used in the present study have been deposited into the NCBI Sequence Read Archive (SRA, https://www.ncbi.nlm.nih.gov/sra/) under the accession number of PRJNA553301 (SRR9656589, SRR9656590, SRR9656591, SRR9656592, SRR9656598, SRR9656597, SRR9656595, SRR9656596 and SRR9656587), in which T0 means 0d; SP1 means 70d and SP2 means 75d. The descriptions of biosamples used in the present study can be found in NCBI BioSample database (BioSample, https://www.ncbi.nlm.nih.gov/biosample) under the accession numbers 12233559, 12233560, 12233561, 12233562, 12233563, 12233564, 12233565, 12233566 and 12233567. All the supporting data are included as additional files.

## Abbreviations

ABA: abscisic acid
Aux: auxin
BR: brassinosteroid
cDNA: complementary DNA
CTK: cytokinin
Ctrl: control
DEGs: differentially expressed genes
DEPs: differentially expressed proteins
ET: ethylene
FDR: false discovery rate
FPKM: fragments per kilobase of transcripts per million mapped
GA: gibberellin
GO: Gene Ontology
IAA: Indole-3-acetic acid
JA: jasmonic acid
KGGG: Kyoto encyclopedia of genes and genomes
MRM: multiple reaction monitoring (MRM)
SA: Salicylic acid
