# Supplementary figures and images for: Integrative omics analysis on phytohormones involved in oil palm seed germination
Source: BMC Plant Biol. 2019 Aug 19;19:363. doi: 10.1186/s12870-019-1970-0 (PMC6700987; doi:10.1186/s12870-019-1970-0)

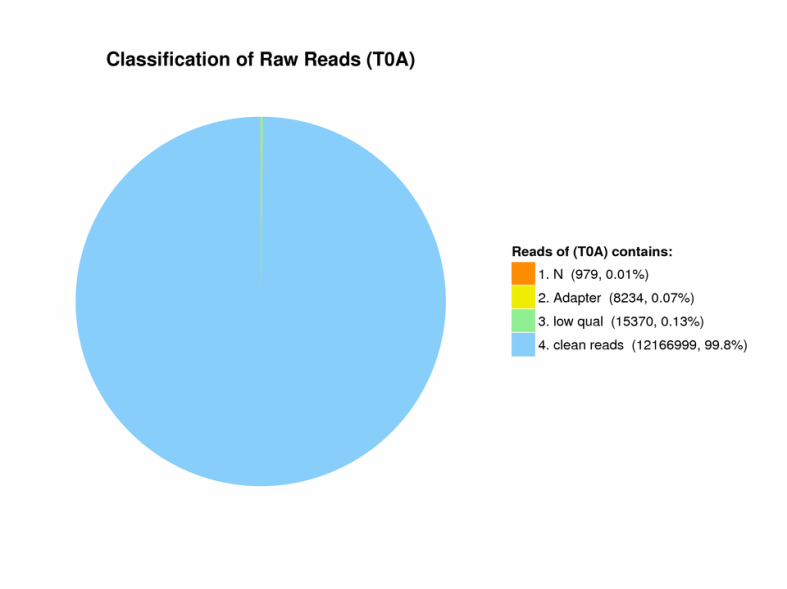

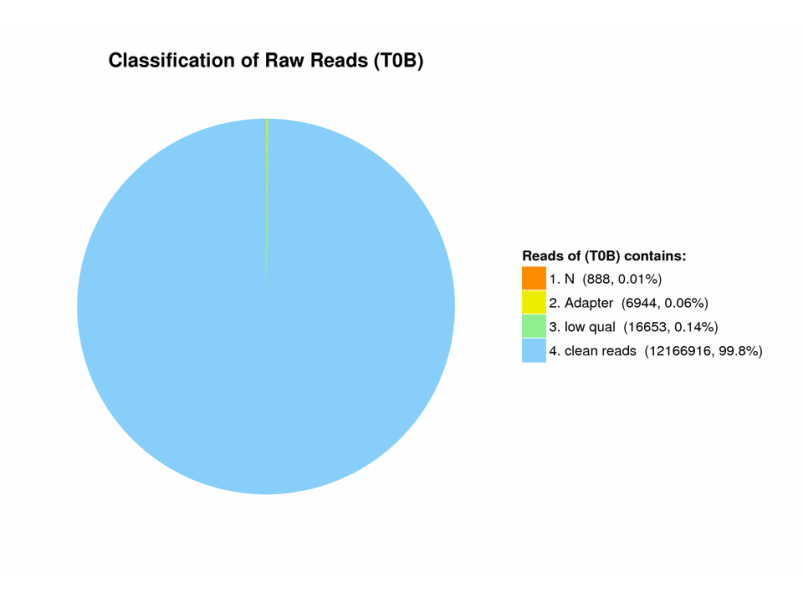


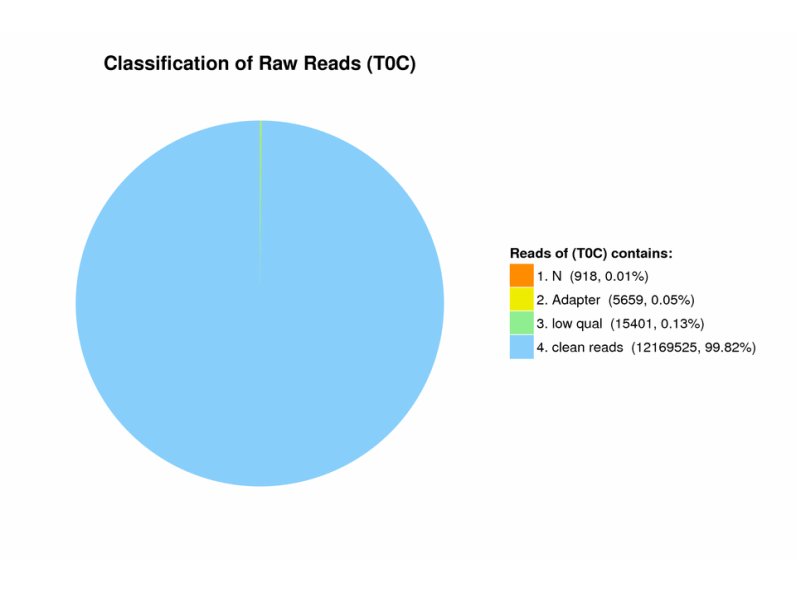

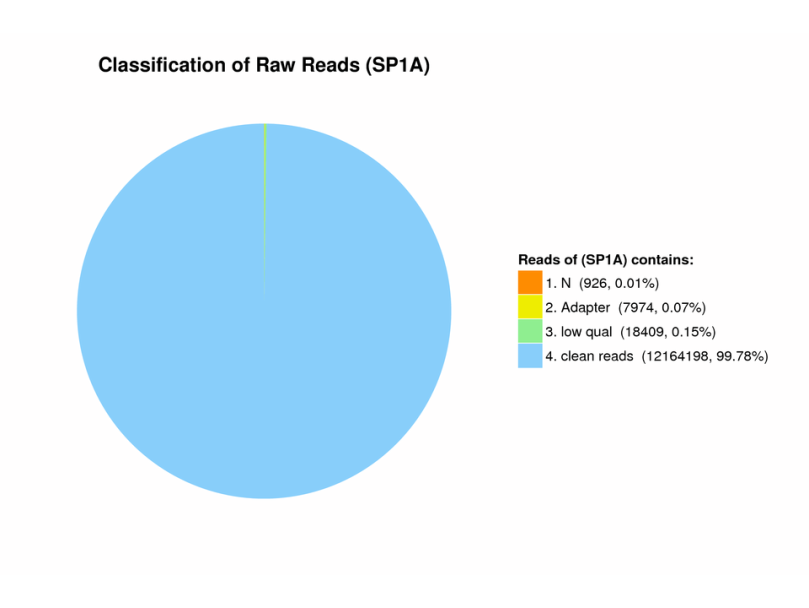


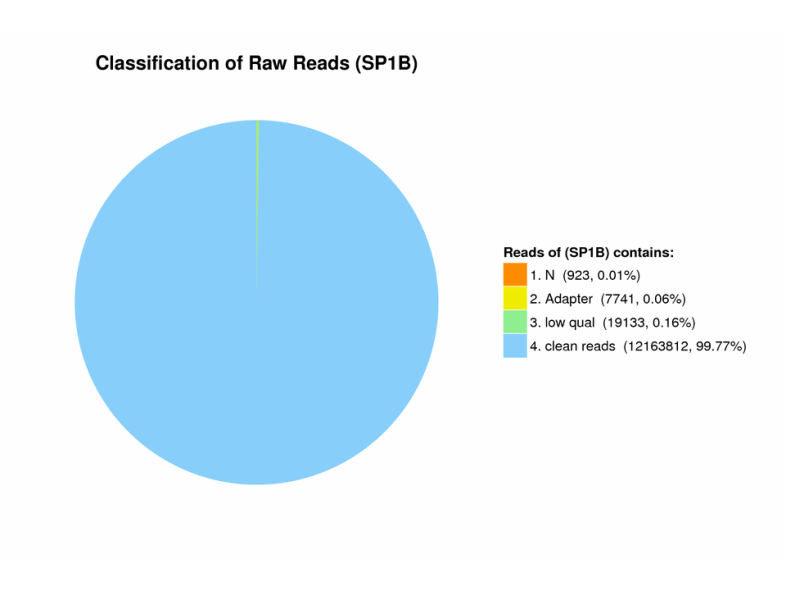

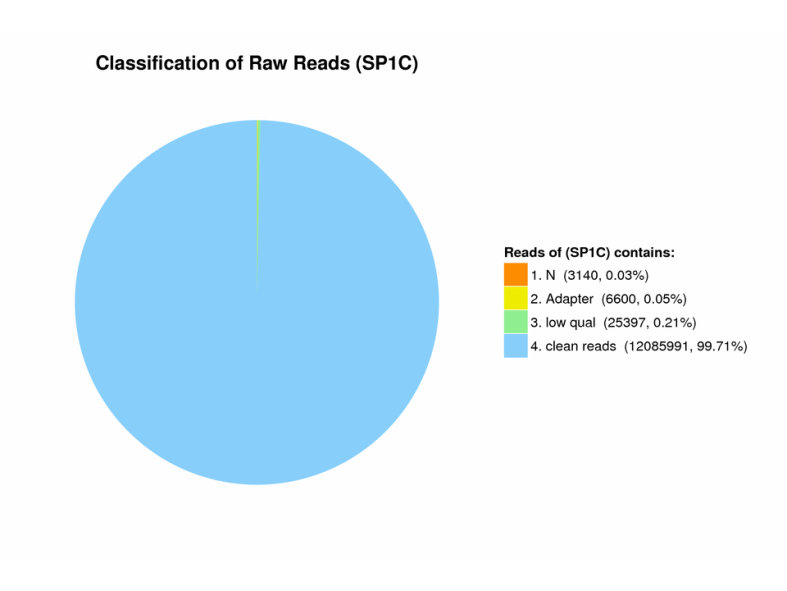


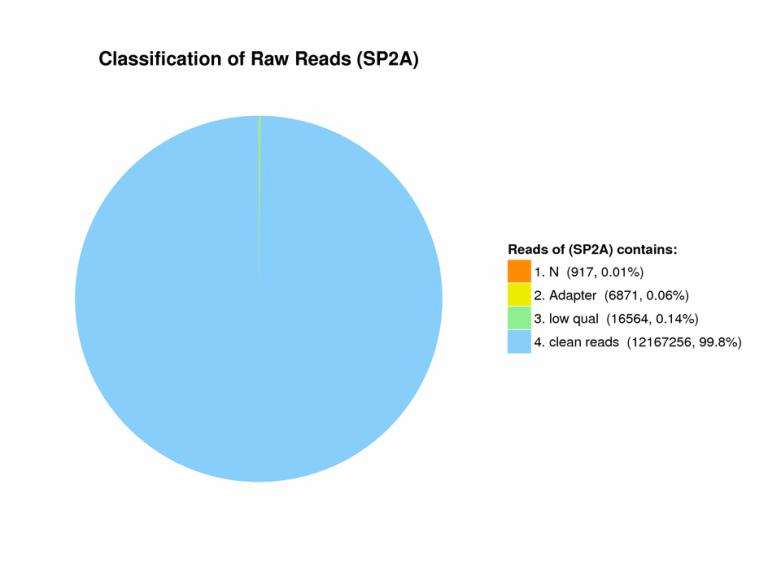

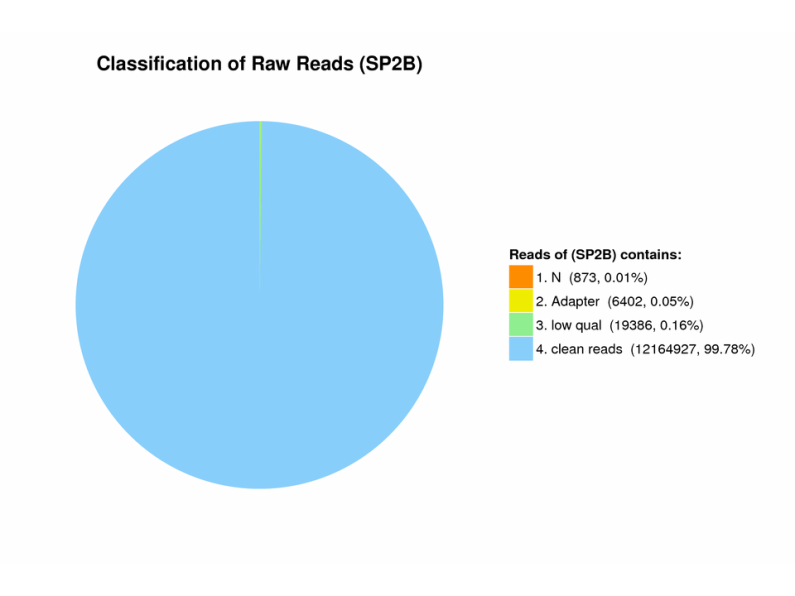


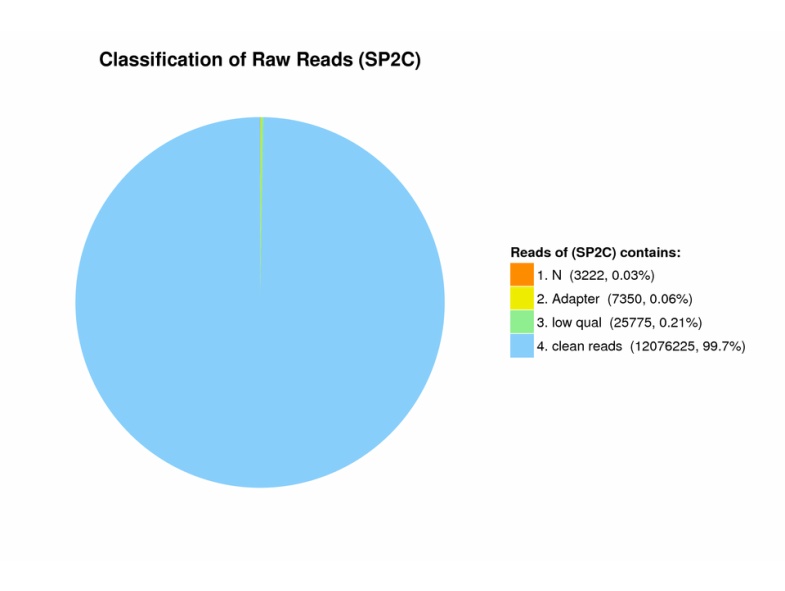


**Figure S1. Quality assessment of reads.** T0: 0d; SP1: 70d; SP2: 75d. **“**A, B and C” represents 3 replicates**.**

Supplement: Supplementary file 1 — Figure S1. Quality assessment of reads. (DOCX 498 kb) [file 12870_2019_1970_MOESM1_ESM.docx]

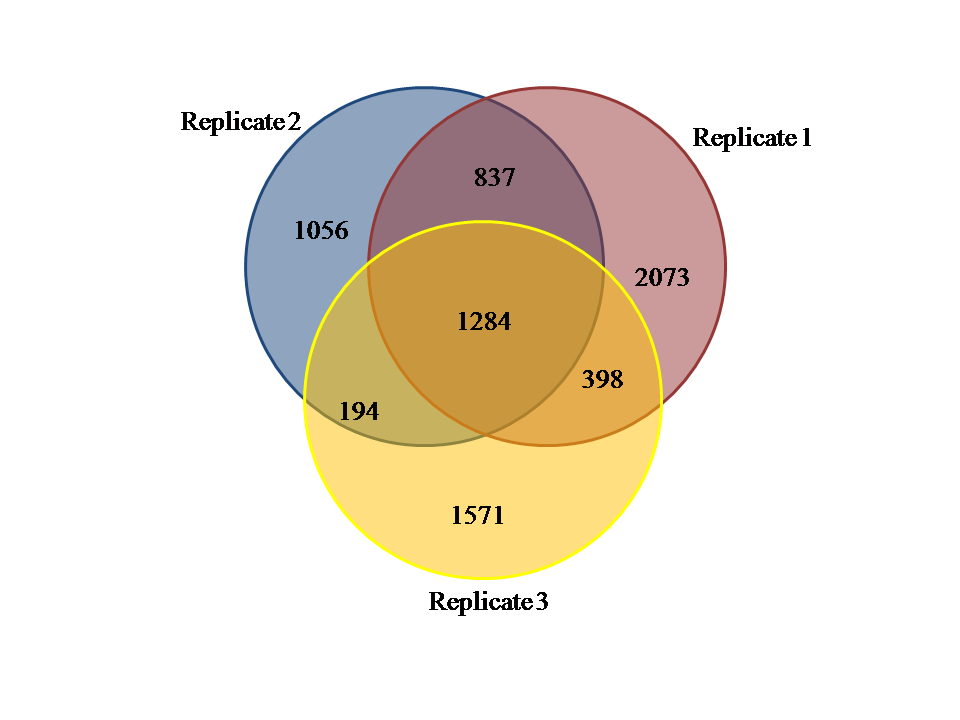


**Figure S2.** **Venn diagram of identified proteins in three biological replicates.**

Supplement: Supplementary file 8 — Figure S2. Venn diagram of identified proteins in three biological replicates. (DOCX 181 kb) [file 12870_2019_1970_MOESM8_ESM.docx]
