# Supplementary material for: Integrative omics analysis on phytohormones involved in oil palm seed germination
Source: BMC Plant Biol. 2019 Aug 19;19:363. doi: 10.1186/s12870-019-1970-0 (PMC6700987; doi:10.1186/s12870-019-1970-0)
Supplement: Supplementary file 10 — Figure S3. Top 10 KEGG pathway enrichment of DEGs. Red ribbons indicate the links between 0d-Vs-70d and top 10 pathways while orange ribbons indicate the link between 0d-Vs-75d and top 10 pathways. The number of DEGs in each pathway was represented by the thickness of the ribbons. (DOCX 429 kb) [file 12870_2019_1970_MOESM10_ESM.docx]

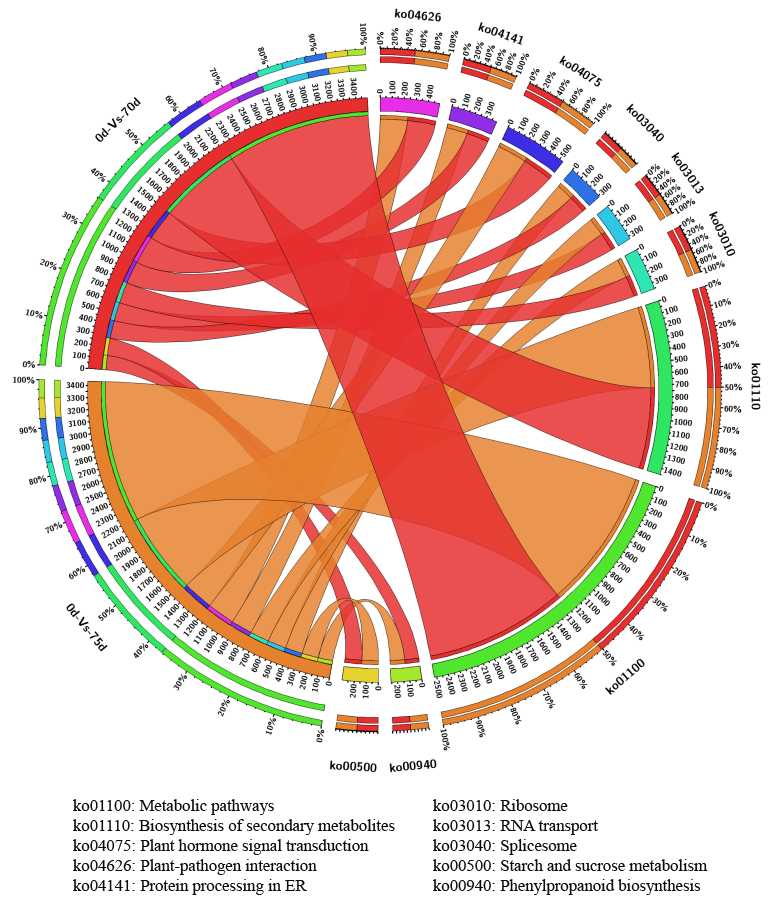


**Figure S3.** **Top 10 KEGG pathway enrichment of DEGs.** Red ribbons indicate the links between 0d-Vs-70d and top 10 pathways while orange ribbons indicate the link between 0d-Vs-75d and top 10 pathways. The number of DEGs in each pathway was represented by the thickness of the ribbons.
