# Supplementary material for: Integrative omics analysis on phytohormones involved in oil palm seed germination
Source: BMC Plant Biol. 2019 Aug 19;19:363. doi: 10.1186/s12870-019-1970-0 (PMC6700987; doi:10.1186/s12870-019-1970-0)
Supplement: Supplementary file 12 — Figure S4 Top 10 enriched GO categories of differential proteins in (A) 0d-Vs-70d (B) 0d-Vs-75d as compared with all identified proteins. (DOCX 102 kb) [file 12870_2019_1970_MOESM12_ESM.docx]

**A**

**B**

**Figure S4.** **Top 10 enriched GO categories of differential proteins in (A) 0d-Vs-70d (B) 0d-Vs-75d as compared with all identified proteins.** All identified proteins are given as reference set and differential proteins detected between two samples are given as test set. GO categories of test set were compared with that of reference set.
